# Staff views of a hospital at home model implemented in a Scottish care setting

**DOI:** 10.3934/publichealth.2021036

**Published:** 2021-06-09

**Authors:** Katherine Karacaoglu, Calum F Leask

**Affiliations:** 1 Aberdeen City Health and Social Care Partnership, Marischal College, Broad St, Aberdeen, UK; 2 Health Intelligence Department, NHS Grampian, Eday Rd, Aberdeen, UK

**Keywords:** hospital at home, geriatrics, community, implementation, evaluation

## Abstract

**Purpose:**

Demographic and financial challenges mean prioritising a shift in healthcare provision from acute to community settings. One well-evidenced model encapsulating this is ‘hospital at home’, however limited research has examined staffs' views on its implementation, which may inform service development and increase job satisfaction. The aim within was to explore the staff perspective of implementing a ‘hospital at home’ model in a Scottish care setting which can inform service provision and ultimately increase job satisfaction.

**Methods:**

The ‘Acute Care @ Home’ (AC@H) service had a multi-disciplinary team. Referrals were predominantly received from a geriatric hospital ward. Inclusion criteria were older adults with geriatric syndromes and who required care input for a duration between one to seven days. In-depth staff interviews (N = 13) were conducted and analysed thematically to understand barriers and facilitators to implementation. These were supplemented with questionnaires assessing constructs of interest including training, communication and overall satisfaction.

**Results:**

Several themes urged from our study: inter-team and intra-team collaboration, service development and operation, and scaling considerations. High job satisfaction was reported (mean score 73%), particularly due to a perceived non-hierarchical team structure and inclusive management style. Staff attributed positive outcomes through better identifying patients' needs at home compared to in hospital. Continuity of care facilitated rapport building. Recruitment challenges restricted the acuity and volume of patients the team were able to care for.

**Conclusions:**

This qualitative methodology could be useful for future implementation of intermediate care resources for the future health and care system building. Patient assessments at home, as opposed to in hospital, in conjunction with care continuity by staff, may mitigate against hospital risks and better facilitate reablement. Where recruitment challenges are present, agile models of care delivery should be considered.

## 1. Introduction

The global population is facing unprecedented change, with the proportion of those over 80 years predicted to triple by 2050 [1]. This demographic shift is placing considerable demand on acute services, with 58.9% of unscheduled hospital admissions from over 85s in England [2]. The consequential financial implications have resulted in a cumulative deficit from NHS trusts and Foundation trusts of £791m in 2016–2017 [3]. It is recognised locally [4] and nationally [5] that current models of health and social care delivery are unsustainable, and alternative initiatives to shift the balance of care from acute to community settings are required. Home care schemes, including intermediate care at home and home-based primary care (HBPC) models, amongst others, have seen a resurgence due to the increase in the number of older populations with multiple chronic conditions and high needs, including frailty and multimorbidity [6]. These changes have been accelerated by the COVID-19 pandemic, partly due to the disproportionate risks to this cohort, resulting in an increase in virtual consultations and intermediate care at home to protect patient safety [7].

One such model of care is ‘hospital at home’ (H@H), characterised by acute care provision, traditionally delivered in a hospital setting, in an individual's own home [8]. A multi-disciplinary team (MDT) provide active treatment for a limited period, typically 1–7 days. The model is generally operationalised through one of two pathways: early supported discharge (referral received from an acute ward allowing a patient to receive the final part of their acute care at home) and admission avoidance (referral received through the patient's GP to prevent hospital admission) [9]. Previous research has demonstrated that the H@H model lessened inpatient length of stay by 62% [10], shortened overall intervention length [11] and can reduce costs by 41% through healthcare usage efficiencies (e.g. few laboratory orders and imaging) [12]. A recent multi-site randomised control trial described reduced likelihood of patients living in an institutional setting at 6 month follow-up [13], however, there is still limited evidence of staff views of this model of care delivery [14]. Of the literature, one study focused on GP satisfaction [15] whilst another explored the impact of the model on GP workload [16], as opposed to those delivering the service. A recent study reports high healthcare professional satisfaction rates (98%), however provides no insight into why or how this satisfaction was achieved [17].

Ascertaining staff perceptions can provide valuable insight to operational barriers and facilitators through their lived experience of service delivery [18]. This is a key mechanism through which feasibility can be understood, particularly important when implementing an initiative within a new context [19]. Further, engaging staff in decisions regarding service development is associated with improved staff satisfaction and reduced turnover rates [20]. This is of particular importance considering recruitment and retention challenges of health professionals both locally [21] and globally [22].

The aim of this study is to understand staff views of implementing a H@H model in a Scottish care setting.

## 2. Methods

### 2.1. Service design

The Acute Care at Home (AC@H) service was part of a transformation programme to redesign local services. The MDT were based in a community hospital and consisted of 1 x Advanced Nurse Practitioner (ANP), 1 x Physiotherapist (PT), 1 x Occupational Therapist (OT), 5 x Health Care Support Workers (HCSWs), 2 x Pharmacy Technicians (PTech, covering 0.5wte post) and overseen by a Team Leader (TL). Whilst the service did not have exclusive use of a physician, it received professional support and clinical guidance from a Consultant Geriatrician (Geriatrician) working on a discharging acute ward. AC@H predominantly utilised the early supported discharge pathway. Referrals were received from a Geriatric hospital ward and patients were typically in receipt of care for between 1–7 days, rather than remaining in hospital which would have occurred previously. The AC@H team predominantly provided rehabilitation support from nursing, physiotherapy and occupational therapy perspectives. The most frequently reported interventions that the AC@H team carried out included patient assessments, observations and reviews (35%), referrals and signposting to other services (24%), equipment provision (e.g. raised toilet seats, 16%) and personal care (11%). Inclusion criteria were: over 75 years with geriatric syndromes and either requiring assistance or managing independently with personal care and where support was required during their acute need (or following recovering of an acute condition).

### 2.2. Data collection and analysis

Semi-structured interviews (N = 13) were conducted with staff members who delivered or managed the service, following a topic guide to stimulate discussion around their experience working in this service. Topics discussed included: 1) overall experience; 2) enablers to service implementation; 3) barriers to implementation; and 4) future development considerations. Interviews were audio recorded and lasted no longer that 60 minutes. Field notes were taken by researchers during the interview for reference during data analysis.

Interviews were supplemented with a questionnaire comprised of numerous constructs of interest that may impact on service implementation. These included: perceived development opportunities; workload; team working and communication (Appendix A). Constructs were chosen as they combined key process evaluation constructs relating to staff satisfaction evident in the literature [23] and locally agreed outcomes of interest.

### 2.3. Analysis

Audio recordings were transcribed verbatim and were analysed thematically, using NVivo software Version 11 (QSR International, Doncaster). Thematic analysis is a method of identifying patterns in data around a specific area of interest, in this case, staff experience of working in the AC@H team [24]. Data analysis using this approach, described by Braun and Clarke, follows a six step frameworks: 1) data familiarisation; 2) initial code development; 3) searching for themes; 4) reviewing of themes; 5) defining themes and 6) results write up [25]. Authors independently analysed the data then compared findings and made adaptations, where necessary, until agreement was reached. Disagreements were settled by consulting with a third, external researcher.

## 3. Results

### 3.1. Service overview

An overview of the first six months of operation and demographic profile of patients are visible in Table 1. The most commonly reported referral reasons included mobility issues/assessment (29%), activities of daily living concerns (17%) and other assessments (e.g. functional assessment, 15%). Following an AC@H episode of care, 81% of patients were discharged home and 9.5% required a hospital admission. The remaining were discharged to other locations e.g. supported living. At 90 days following an AC@H admission, 79% of patients were living at home or in a community setting, 11% were deceased and 10% were in hospital.

**Table 1.** Characteristics of AC@H caseload.

Characteristic	Total
Caseload, N	84
Female, N (%)	54.8 (45.2)
Age, mean (range)	86.2 (67–102)
SIMD Scores N (%)	
1	9 (10.7)
2	19 (22.6)
3	6 (7.1)
4	10 (11.9)
5	31 (36.9)
Not reported	9 (10.7)
Caseload days, mean (range)	5.2 (1–17)
Number of visits per patient (mean, range)	5 (1–21)

Note: NB: SIMD = Scottish index of multiple deprivation with scores from 1 (most deprived) to 5 (least deprived).

### 3.2. AC@H staff satisfaction

Table 2 displays staff satisfaction questionnaire responses. Staff appeared to be satisfied in their role, in particular feeling supported by management and indicated effective communication with AC@H colleagues.

**Table 2.** Staff satisfaction questionnaire scores (N = 10).

Questionnaire components	Mean Score
Support	3.6
Training	3.3
Development	2.8
Communication	3.6
Workload	3.1
Progression	2.5
Recognition	3.6
Teamwork	3.2
Systems	2.9
Overall satisfaction	7.3

Note: NB: Questionnaire components scored on a 5-point Likert scale, whilst overall satisfaction was scored on a 10-point Likert scale.

### 3.3. AC@H staff experience

Interviewee characteristics are displayed in Table 3. To ensure participant anonymity, the ANP, PT and OT are collectively defined as “Advanced Practitioners (APs)”, whilst the TL, Geriatrician and Senior Service Manager are collectively defined as “Management”.

**Table 3.** Characteristics of interviewed AC@H staff (N = 13).

Participant ID	Sex (M/F)	Experience (yrs.)	Role
P1	F	>10	Advanced Practitioner
P2	F	>10	Advanced Practitioner
P3	F	>10	Advanced Practitioner
P4	M	2–5 years	Health Care Support Worker
P5	F	>10	Health Care Support Worker
P6	F	>10	Health Care Support Worker
P7	F	6–10 years	Health Care Support Worker
P8	F	>10	Health Care Support Worker
P9	F	>10	Pharmacy Technician
P10	F	>10	Pharmacy Technician
P11	F	-	Management
P12	M	-	Management
P13	F	-	Management

### 3.4. Themes

Four themes emerged: 1) Service development (strategies to facilitate team functioning); 2) Relationships (within and out with the team); 3) Service operation (service operation characteristics); and 4) Scaling considerations (future development challenges) (Table 4).

**Table 4.** Themes and sub-themes derived from AC@H team interview analysis.

Theme	Sub-theme
Service development	Upskilling
	Resources
Relationships	Inter-team collaboration
	Intra-team collaboration
Service operation	Care Delivery
	Satisfaction
	Agility
Scaling considerations	Medical input solutions
	Operational adjustments

#### 3.4.1. Service development

**Upskilling**—Staff reported high satisfaction with training received which focused on frequently utilised skills and in addition APs enrolled in a Master's degree in Clinical Practice. Staff felt empowered by management to seek their own development opportunities: *“Training wise everything is available to you...you just need to ask [name] or one of the senior members of staff”* (P5, HCSW). However, a tension existed between training uptake and sufficient staff available for service operation, particularly due to limited APs within the team: *“...the other two [APs] have assignments due in tomorrow and have been off all week, so this has a massive effect on how many patients we can take into the service because there is only me here to assess them”* (P3, AP).

**Resources**—The temporary team office brought challenges, including being overcrowded and not conducive to productivity: *“...it is a small room with a lot of people cramped in to it ... there are constant interruptions while you are there”* (P1, AP). Solutions were sought to cope with this challenge including keeping busy outwith the office and booking meeting rooms for space to concentrate. Staff remained optimistic about the move to their permanent location: *“We will all have our own space. It is just a lot bigger, it is a lot nicer, it's a lot better”* (P7, HCSW).

#### 3.4.2. Relationships

**Inter-team collaboration**—Participants described the presence of a positive team relationship where staff felt supported and valued regardless of their position: *“Staff overall get on, it's a great team. There is no like, you know, hierarchy or things like that. Everyone is treated as an equal”* (P5, HCSW). Strong team dynamic appeared to be facilitated by high satisfaction with management staff due to their personable qualities: *“She is really dynamic, very positive and you can see her passion for the whole project and wanting to drive it forward”* (P1, AP). Management were described as transparent and involved staff in all aspects of service development, including decision making around patient care: *“any changes with the patient, we have a meeting and discuss the patient and we're asked for feedback once we've seen the patient so I do feel like we are really included”* (P8, HCSW).

**Intra-team collaboration**—Participants described positive relationships with acute and community teams, particularly as staff had developed relationships prior to taking up their posts and as the AC@H team were co-located with other services in their temporary location: *“we have the community OTs, PTs, Dietitians, Speech and Language. I think there are a lot of services, in fact the services that we would refer on to apart from Care Management are within the building. So from that point of view it has been great”* (P9, PTech). Some participants felt communication may become more challenging in their permanent location: *“It will involve a lot more phone calls and things I would imagine once we move away”* (P2, AP).

#### 3.4.3. Service operation

**Care delivery**—Due to staff upskilling requirements, reablement care was predominantly provided: *“...assisting with personal hygiene, meal prep, bed prep, you know some medication prompt, we have had some physiotherapy where we have been going in and doing a bit of exercise with them...”* (P6, HCSW). Positive improvements in functional status were described with staff enabling patients to live as independently as possible: *“At the end of the seven days the person is actually back to their baseline and we are actually able to pull out”* (P2, AP). More complex patients began entering the service and showed improvements in acute symptoms: *“Chest infections...we had seen a big improvement from when we started from day one to seeing them on their last day”* (P4, HCSW). Care provided in patients' own home reduced concerns of hospital acquired infections: *“...the risks, they are exposed to more bugs and germs [in hospital], they are at a higher risk for their health”* (P1, AP).

Service characteristics that functioned well included assessment of patients in their own home, as opposed to in hospital, which was seen as more effective in identifying actual patient need and in turn allowed more appropriate adaptations to be put in place to facilitate independent living: *“You pick up on things, I think, when we go in to the home environment that would maybe not be picked up in the hospital...maybe move round their furniture, maybe different equipment that we could maybe be using in their own house that is maybe not in situ [in place] that would make their life easier”* (P6, HCSW). The presence of an MDT, in conjunction with newly developed pathways, led to reported efficiencies including rapid access to blood test results and equipment provision. In addition, participants described the ability to build a rapport with patients due to the small team size: *“They all like continuity, they like the same person going in...they look forward to you coming”* (P8, HCSW).

**Satisfaction**—Staff received predominantly positive service feedback, in particular being able to receive care in their own home, with benefits including being able to have support networks close-by: *“At least when they are in their home environment they are in their comfort zone. Family, friends and all that have a lot easier access if they have those people round about them”* (P1, AP). Participants reported that patients found having the team to support them at a critical time, transitioning home from hospital, was important in building confidence: *“they just feel relieved, more secure, comfortable realising that they have not just been put out of hospital and abandoned. They have been put out and we are coming in and making sure that they are settled and that you are alright”* (P10, PTech).

**Agility**—Operational modifications were required to address unexpected contextual challenges, in particular being unable to recruit a Geriatrician, resulting in limited acute service admissions. Consequently, the model shifted its focus from clinical care provision to enablement focused: *“it has been away from that kind of disease focused management or very medical kind of modelling, particularly because we have no medic leading”* (P2, AP). Referrals were accepted from an acute geriatric hospital ward once a Geriatrician had ensured the patient was medically fit: *“...we ended up going to a more of a rapid supported discharge type thing...at least then we would have control over the patients being medically stable so that we knew they would not be requiring huge amounts of our input that we couldn't necessarily provide”* (P13, Management).

#### 3.4.4. Scaling considerations

**Medical input solutions**—The acuity of patients was limited by challenges in recruiting a Geriatrician. Potential options thought to address this were to build upon GP expertise and to increase skilled AP input: *“we just keep the GP as a responsible clinician but they have input from PA's [Physician Associates] or training GP, so we are exploring all of those possibilities at the minute. With skilled ANPs or APs I think is as good a concept, as long as we make sure it is safe and there is clear governance structures within that...it doesn't have to be a Geriatrician”* (P11, Management). There was potential described for the responsible clinician to support in upskilling APs, along with formal training, ultimately leading to APs increasing their caseload responsibility safely: *“because there is a confidence between the medic, could that be the Consultant or the GP with the team members, that there is less engagement between them as there is a confidence that has been built there so there is a need for a bit of supervision in there in checking but you are getting to know what that individual is doing”* (P12, Management).

**Operational adjustments**—An expansion consideration described was to broaden the referral pathway to include acute departments and GP practices: *“It will be very slow until it feeds into the GP practices. We could take a lot of load from them if they meet us half way”* (P8, HCSW). Operational hours were also seen as insufficient and a more flexible service to suits to needs of patients necessary: *“You know you are taking on a sick patient, I mean they do not stop being sick at 4 o'clock or on a Friday”* (P2, AP).

## 4. Discussion and conclusions

The aim of this study was to understand staff views of implementing a H@H model in a Scottish care setting, an area where evidence is limited [13]. Staff members lived experience of service implementation can provide a valuable perspective on operational barriers and facilitators [18] and can determine feasibility for those delivering the service within new contexts [19]. Several key themes urged from our study: inter-team and intra-team collaboration, service development and operation, and scaling considerations.

The AC@H team reported high job satisfaction, (mean score 73%), a value 5.1% higher than the national average for NHS Scotland staff [26]. This appeared to be influenced by inter-team collaboration including the perceived lack of within-team hierarchy, and supportive management staff (mean score 72%) who empowered colleagues through inclusive decision-making and autonomy. A management style which is participatory and inclusive has been shown previously to increase employee engagement and reduce burnout [20]. Additionally, staff autonomy to adjust service provision to suit individual needs has been demonstrated in a local community model of care, to be integral to delivery of high quality patient care [27],[28]. Considering healthcare recruitment and retention challenges globally [29], coupled with the association between low job satisfaction and staff turnover, implementing an inclusive management style may not just ensure adequate staff provision, but improve collaboration and facilitate professional development [20],[30].

Staff highlighted advantages of carrying out patient assessments at home, as opposed to hospital, to more accurately identify support required for individuals to live independently. Environmental assessment, specifically by OTs, has been shown to significantly reduce incidence of falls, as additional considerations can be identified not previously highlighted in hospital [31]. This aligns with other HBPC models which describe benefits in the ability to identify home environment factors which could be overlooked at a primary or secondary care appointment (e.g. fall hazards, an empty fridge or family stressors) and in the facilitation of stronger relationship building between patients and healthcare providers [6]. Further, staff reported positive patient feedback regarding continuity of care, which facilitated relationship building between individuals. This is congruent with other integrated community care models where continuity of care was a key, enabling mechanism identified from a patient perspective during service implementation [29]. Consistency in staff providing care has been shown not solely to increase patient and staff satisfaction rates, but also to improve patient outcomes [32], treatment adherence [33] and reduce resource utilisation (including prescription costs and hospital admissions) [34]. If it is safe enough to transition patients from hospital to community settings, the ability to assess patients in their own environment, in conjunction with continuity of care by staff, may mitigate risks associated with hospital admissions (such as infections) and instead facilitate reablement and increase patient satisfaction.

The service was challenged by lack of recruitment to senior clinical roles, specifically a Geriatrician and, the APs when recruited, did not possess the relevant qualifications to deliver acute care provision. Geriatrician recruitment challenges are evident internationally [35], along with numerous other clinical roles, including other physicians, nurses and midwives [36]. Consequently, this limited the volume and acuity of patients able to enter the service. The recruitment challenges coupled with the small team size, particularly only one of each AP, created a tension between staff training completion and sufficient cover for service operation. This was particularly apparent whilst APs were undergoing advanced clinical training during service operational hours. As such, alternative strategies to provide the medical cover required to develop the service, should be explored. A potential solution identified through staff interviews included recruitment of a GP, as opposed to a Geriatrician, as service clinical oversight. Supervisory models of care delivery where sufficiently upskilled health professionals can be delegated tasks, usually carried out by a physician, and under the supervision of a physician (including GPs or Geriatricians), is a model which has shown to effectively and safely increase service capacity [37],[38]. To tackle recruitment challenges, models of care should consider agile and adaptable in their delivery.

There are some limitations to consider. Due to the acuity of patients, it is likely that there may be additional mechanisms to consider as the complexity of patients increases that have not been fully explored here. Secondly, these findings could have been further supplemented by collecting data from other groups of interest, for example unpaid carers, who are an under researched group in this area [14]. However, this was not possible due to time and resource constraints, therefore it is recommended that future research explores unpaid carers' perceptions of this service.

In summary, a management style which is non-hierarchical and inclusive in decision making appears preferable to staff and should be considered for increased job satisfaction and to prevent increased turnover rates. In circumstances where patients can be transferred home safely, assessments carried out in a patient's own home, as opposed to a hospital setting, in combination with continuity of care by staff, may reduce hospital associated risks and better facilitate the reablement process. Lastly, where recruitment challenges are identified, agile and evidenced based care delivery models should be considered to address these. These findings could be beneficial in directing health and care system development and as a resource to support intermediate care implementation.
