# Supplementary material for: Staff views of a hospital at home model implemented in a Scottish care setting
Source: AIMS Public Health. 2021 Jun 9;8(3):467–78. doi: 10.3934/publichealth.2021036 (PMC8334636; doi:10.3934/publichealth.2021036)
Supplement: Supplementary file 1 [file publichealth-08-03-036-s001.pdf]

AIMS Public Health

AIMS Public Health, 8(3): 467–478.
DOI: 10.3934/publichealth.2021036
Received: 12 April 2021
Accepted: 24 May 2021
Published: 09 June 2021

http://www.aimspress.com/journal/aimsph

Research article

Staff views of a hospital at home model implemented in a Scottish care setting

Katherine Karacaoglu[1,2] and Calum F Leask[1,2,*]

[1] Aberdeen City Health and Social Care Partnership, Marischal College, Broad St, Aberdeen, UK
[2] Health Intelligence Department, NHS Grampian, Eday Rd, Aberdeen, UK

* **Correspondence:** Email: CLeask@aberdeencity.gov.uk; Tel: +01224558649.

Appendix A: AC@H Staff Satisfaction Questionnaire

1. What is your occupation? (*tick one option only*)

Geriatrician ☐

Advanced Nurse Practitioner ☐

Physiotherapist ☐

Occupational Therapist ☐

Health Care Support Worker ☐

Pharmacy technician ☐

Other (*please specify*): ____

2. How many years' experience do you have working in either health or social care?

<2 years ☐

2–5 years ☐

6–10 years ☐

>10 years ☐

3. To what extent do you agree with the following statements (tick one box only):

Construct	Question	Strongly disagree	Disagree	Agree	Strongly agree
Supported	*I feel supported by AC@H management staff*				
Training	*I am provided with all necessary training to do my job*				
Development	*I have adequate opportunities to develop my professional skills*				
Communication	*I feel I can easily communicate with members from all levels of the team*				
Workload	*The amount of work I am expected to finish each week is reasonable*				
Progression	*I am satisfied with my chances for promotion*				
Recognition	*I am appropriately recognised when I perform well at my regular work duties*				
Teamwork	*My co-workers and I work well together*				
Systems	*The IT systems I use to do my job are fit for purpose*				
Satisfaction	*How would you rate AC@H as a place to work on a scale of 1 (the worst) to 10 (the best)?*	1 2 3 4 5 6 7 8 9 10<br>Worst Best			

4. Please let us know if you have any additional comments below:

____

____

____

Thank you for taking the time to complete this questionnaire

AIMS Press

© 2021 the Author(s), licensee AIMS Press. This is an open access article distributed under the terms of the Creative Commons Attribution License (http://creativecommons.org/licenses/by/4.0)
